# IV BCG Vaccination and Aerosol BCG Revaccination Induce Mycobacteria-Responsive γδ T Cells Associated with Protective Efficacy against *M. tb* Challenge

**DOI:** 10.3390/vaccines11101604

**Published:** 2023-10-17

**Authors:** Alexandra L. Morrison, Charlotte Sarfas, Laura Sibley, Jessica Williams, Adam Mabbutt, Mike J. Dennis, Steve Lawrence, Andrew D. White, Mark Bodman-Smith, Sally A. Sharpe

**Affiliations:** 1 Vaccine Development and Evaluation Centre, UK Health Security Agency, Porton Down, Salisbury SP4 0JG, UK; 2 Infection and Immunity Research Institute, St. George’s University of London, London SW17 0BD, UK

**Keywords:** BCG, γδ T cells, tuberculosis

## Abstract

Intravenously (IV) delivered BCG provides superior tuberculosis (TB) protection compared with the intradermal (ID) route in non-human primates (NHPs). We examined how γδ T cell responses changed in vivo after IV BCG vaccination of NHPs, and whether these correlated with protection against aerosol *M. tuberculosis* challenge. In the circulation, Vδ2 T cell populations expanded after IV BCG vaccination, from a median of 1.5% (range: 0.8–2.3) of the CD3+ population at baseline, to 5.3% (range: 1.4–29.5) 4 weeks after *M. tb*, and were associated with TB protection. This protection was related to effector and central memory profiles; homing markers; and production of IFN-γ, TNF-α and granulysin. In comparison, Vδ2 cells did not expand after ID BCG, but underwent phenotypic and functional changes. When Vδ2 responses in bronchoalveolar lavage (BAL) samples were compared between routes, IV BCG vaccination resulted in highly functional mucosal Vδ2 cells, whereas ID BCG did not. We sought to explore whether an aerosol BCG boost following ID BCG vaccination could induce a γδ profile comparable to that induced with IV BCG. We found evidence that the aerosol BCG boost induced significant changes in the Vδ2 phenotype and function in cells isolated from the BAL. These results indicate that Vδ2 population frequency, activation and function are characteristic features of responses induced with IV BCG, and the translation of responses from the circulation to the site of infection could be a limiting factor in the response induced following ID BCG. An aerosol boost was able to localise activated Vδ2 populations at the mucosal surfaces of the lung. This vaccine strategy warrants further investigation to boost the waning human ID BCG response.

## 1. Introduction

Tuberculosis (TB) remains a leading cause of mortality due to an infectious disease, with COVID-19 halting progress to reduce the high global burden of TB [1]. In 2021, there were 1.6 million deaths from TB, and an estimated 10.6 million new *M. tuberculosis* (*M. tb*) infections [1]. The only available vaccine against TB is Bacille Calmette-Guerin (BCG), an attenuated form of *Mycobacterium bovis*. The BCG vaccine provides up to 80% protection against the most severe forms of TB, including TB meningitis in children [2]. However, immunity wanes by adolescence, and protection afforded against pulmonary TB in adults ranges widely between studies in different geographical locations, from 0 to 80%. The most recent meta-analysis by Martinez et al. found no protection from BCG in adolescents and adults [3]. Therefore, alternative vaccine strategies are essential.

There are several promising TB vaccines in various stages of the vaccine pipeline [4]. Some of these vaccines aim to replace infant BCG vaccination altogether, whereas others could be provided as a booster when BCG vaccination wanes. However, many candidates have failed to surpass the level of protection afforded with BCG in pre-clinical studies [5], or provide the off-target effects that BCG has in reducing infant mortality against other diseases, notably respiratory viruses with the mechanism of trained innate immunity [6]. An alternative line of investigation is evaluating the effect of BCG revaccination [7,8,9,10], coupled with changing the route of BCG administration [11,12].

Aligning the vaccination route with the primary route of *M. tb* infection by delivering BCG via the aerosol route may provide immunological advantages, as well as removing the requirement for using needles that require highly trained personal and safe disposal. There is evidence from work in the macaque model that primary vaccine delivery via the pulmonary route may be more protective than the intradermal (ID) route [13,14], and also prevent infection [15]. Studies in mice and guinea pigs have found aerosol/intranasal delivery to induce quicker [16] and greater [17,18] immune responses locally in the lungs, in comparison to the ID route, including greater activation of alveolar macrophages [19] after *M. tb* challenge. However, other macaque studies indicate that many immune responses may be delayed after a single primary aerosol BCG vaccination [20], and boosting via this route is a promising alternative [11].

Intravenous (IV) BCG vaccination has been shown to greatly enhance TB protection in non-human primate (NHP) models [11,12,21], but in its current form is unlikely to be approved as a viable vaccination route in humans due to safety concerns. Despite this, IV BCG vaccination provides us with a valuable tool to study correlates of protection and a benchmark against which to test new vaccination strategies. Sharpe et al. used the NHP model of IV BCG to determine that IFN-γ- and TNF-α-producing CD4+ T cells were associated with TB protection [11]. More recently, Darrah et al. found IV BCG induced sterilising immunity in macaques against low-dose challenge, confirming the superiority of this route and the role of antigen-responsive CD4+ and CD8+ T cells in protection [12]. Evidence for these immune cells and cytokines has also been found in humans that control TB [22,23].

In addition to changes in conventional T cells, Darrah et al. also identified an increase in Vγ9Vδ2 T cells in the BAL fluid after IV BCG [12]. γδ T cells are unconventional cells that can bridge between the innate and adaptive immune systems, and the Vγ9Vδ2 (Vδ2 for short) subtype has a unique strategy to rapidly recognise mycobacteria. Bares and Noble found γδ T cells, from patients, controlling TB expanded more in vitro than those from patients not controlling TB [24]. In 2002, Shen et al. provided evidence suggesting Vδ2 cells were involved in a protective BCG vaccination response. They showed that intravenously delivered BCG induced expansion of the Vδ2 cells in the circulation, pulmonary alveoli and intestinal mucosae (4).

We aimed to utilise the macaque IV BCG vaccination and aerosol TB challenge model to confirm the role of Vδ2 cells in the IV BCG immune response, and to determine whether γδ T cell signatures correlated with TB disease protection. We used immunophenotyping and functional flow cytometry panels to characterise the Vδ2 cell response to vaccination, then determined how they may be involved in a protective response and whether this differed to responses defined after ID BCG. Furthermore, we investigated whether an aerosol BCG boost could induce changes to the Vδ2 cells locally that were similar to the IV BCG benchmark.

## 2. Materials and Methods

### 2.1. Animals

**Figure 1:**
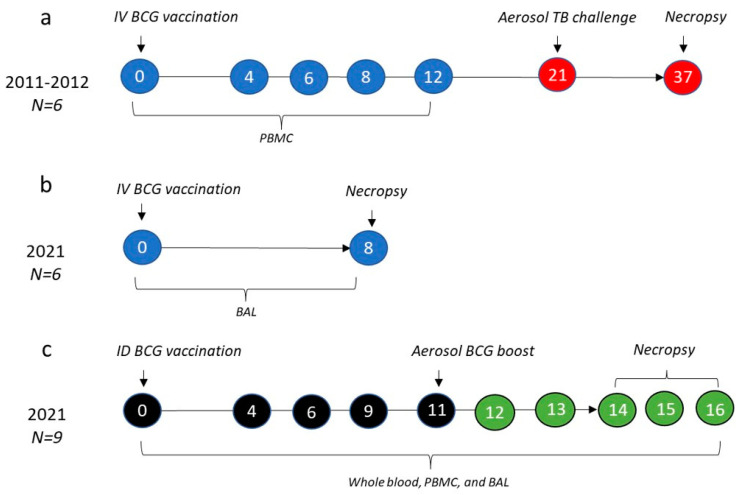
Experimental study design. The data described here are the result of one historic (**a**) and two recent (**b**,**c**) studies. Numbers in circles represent weeks after initial BCG vaccination in each of the studies, with blue circles indicating intravenous (IV) BCG, black indicating intradermal (ID) BCG, green indicating aerosol BCG boost and red indicating an aerosol *M. tb* challenge. Year of study, numbers (N) of rhesus macaques in each study and the sample type collected and included in this paper are described for each study.

A total of 21 rhesus macaques (*Macaca mulatta*) of an Indian genotype sourced from a UK breeding colony were used in this study. All animals were adult males, aged four—12 years. Animals were housed in compatible social groups in accordance with UK Home Office and NC3Rs guidelines, as described previously [11]. All studies and procedures were approved by the UKHSA Establishment Animal Welfare and Ethical Review Committee, Porton Down, UK, and authorised under a UK Home Office project license. Animals were monitored throughout the studies for behavioural or clinical abnormalities, including changes in feeding patterns, respiration rate, body weight, temperature and red blood haemoglobin concentration, as well as a thoracic radiograph for *M. tb*-challenged animals (Figure 1a).

### 2.2. Study Design

Three separate studies were carried out, as outlined in Figure 1. The study described in Figure 1a was part of a larger vaccination route comparison study that has been previously published [11]. All macaques in this study were males and four years of age. Animals in this group were vaccinated intravenously with 1000 μL of Danish strain BCG (SSI, Copenhagen, Denmark) into the femoral vein of the left leg, as previously outlined [11]. Cryopreserved peripheral blood mononuclear cells (PBMCs) collected at 0, 4, 6, 8 and 12 weeks after vaccination were utilised for the assays described here. Figure 1b describes a second study in which six animals were IV BCG-vaccinated (as above) and 8 weeks later, immune analyses were carried out on cells isolated from bronchoalveolar lavage (BAL) samples to provide an understanding of the local immune response at the mucosal surfaces of the lung following IV BCG. All macaques were males and 10 to 12 years of age. PBMCs and other material from this study were also evaluated for concurrent studies, not reported here.

Lastly, in the third study described in Figure 1c, nine animals were vaccinated with 100 μL of Danish strain BCG (SSI, Copenhagen, Denmark) delivered using intradermal injection, then 11 weeks later received an aerosol boost, using approximately 1 × 10^7^ colony forming units (CFU) of aerosolised BCG achieved using an Omron U22 vibrating mesh nebuliser. Immune responses in samples of whole blood and PBMCs were measured after primary vaccination and boosting vaccination. BAL samples were collected for a local immune analysis prior to week 0, and at weeks 9, 12 and 14–16 (at necropsy) post ID BCG. All macaques in this study were males and six years of age.

### 2.3. Tuberculosis Challenge

Animals in the study outlined in Figure 1, a were challenged 21 weeks after IV BCG vaccination with *M. tuberculosis* Erdman strain K 01 (BEI Resources), prepared as previously described [11]. A target dose of 100 CFU delivered to the lungs resulted in an estimated retained dose, calculated from the presented dose by applying retention factors specific for rhesus macaques [25,26,27], that ranged between 64 and 145 CFU, as described fully for each animal in the study by White et al. [11].

### 2.4. Clinical Outcomes

The clinical outcomes following exposure to *M. tb* for the macaques in the study in Figure 1, a have previously been described [11]. The gross pathological changes were scored immediately during the post mortem according to an established system based on number and extent of lesions present in the lungs (lung score), spleen, liver, kidney and lymph nodes (total score) [28]. Thoracic radiographs (SP VET 3.2, Xograph Imaging Systems, Ltd., Tetbury, UK) were acquired and evaluated by a consultant radiologist, with disease burden scored according to a system previously described [11], with the total score termed ‘X-ray score’ here. Magnetic resonance imaging (MRI) was carried out on ex vivo expanded, fixed lungs set in 2% agarose (Sigma-Aldrich, Gillingham, UK) with a 3.0 T 750 MRI Scanner (General Electric Healthcare, Milwaukee, WI, USA). Lung lesions were identified on the MR images, as previously described [11,26,28]. Lastly, a lung consolidation was established via histopathological examination, and scored based on the size, nature and severity of the microscopic lesions [11].

### 2.5. Mononuclear Cell Isolation

Peripheral blood mononuclear cells (PBMCs) were isolated from heparin anti-coagulated blood using standard methods, as previously described [29]. Mononuclear cells were isolated from the bronchoalveolar lavage (BAL) fluid samples, collected using a bronchoscope (Allscope XE30 4 mm flexible bronchoscope; VES, Essex, UK), using standard procedures. In brief, BAL samples were centrifuged at 400 g for 10 min and the pellet was resuspended in RPMI + 10% FCS, then filtered through a 70 μm cell filter to remove debris and mucous. The filtrate was then treated with an ACK red blood cell lysis buffer (Thermo Fisher Scientific, Swindon, UK) for 5 min, before washing. Viable cell counts were performed using trypan blue before use in downstream assays.

### 2.6. Surface Staining and Flow Cytometry

A surface staining antibody cocktail was developed using the following fluorochrome conjugated antibodies prepared in a brilliant stain buffer (BD Biosciences, Wokingham, UK): CD3 AF700, CD195 (CCR5, 3A9) PerCP-Cy5.5 (both BD Biosciences, Wokingham, UK); TCR Vγ9 (7A5) FITC, TCR Vδ1 (TS8.2) PE-Cy7 (both Life Technologies, Paisley, UK); NKG2D APC, CD27 BV711, CD69 BV510, CX3CR1 BV421, PD-1 BV605, CD103 BV785 (all BioLegend, London, UK); CD45RA APC-Vio770 (Miltenyi Biotec, Bergisch Gladbach, Germany); and TIM3 PE (Bio-Techne, Abingdon, UK). Amine-reactive Live/Dead Fixable Red viability cell stain (Life Technologies, Paisley, Renfrew, UK) was also included to enable separation of non-viable cells. Antibodies were titrated for use with mononuclear cells as well as whole blood.

For evaluation, cryopreserved PBMC (Figure 1a) samples were thawed in batches, such that analyses of cells collected at all timepoints from individual animals were carried out on the same occasion. Samples were thawed rapidly at 37 °C, washed twice in RPMI  +  1 U/mL DNase (Sigma-Aldrich, Gillingham, UK) and incubated for 2 h at 37 °C + 5% CO_2_ in RPMI + 10% FCS (R10) at a concentration of 1–2 × 10^6^/mL.

For surface staining of incubated PBMCs or freshly isolated BAL mononuclear cells, samples were washed with a FACs buffer (PBS  +  1% FCS), counted and 1 × 10^6^ cells were incubated at room temperature with the surface staining antibody cocktail above for 30 min in the dark. Then, samples were washed twice with the FACs buffer and fixed with a final concentration of 4% paraformaldehyde (Sigma-Aldrich, Gillingham, UK).

BD Compbeads (BD Biosciences, Wokingham, UK) were labelled with the above fluorochromes for use as compensation controls. Cell data were acquired using a five-laser LSRII Fortessa instrument (BD Biosciences, Wokingham, UK) and data were analysed using FlowJo software (version 10.7.2, BD Life Sciences, Ashford, AL, USA). Appropriate gating strategies were used to identify the lymphocyte population, exclude doublet events and gate on viable cells only. Vδ1 and Vδ2 subtypes were delineated using the expression of CD3, Vδ1 and Vγ9; their activation status using CD69 and NKG2D; their memory phenotype using CD45RA and CD27; their homing marker expression using CCR5, CX3CR1 and CD103; and their expression of the regulation/exhaustion markers using PD-1 and TIM3. Graphpad Prism (version 9, GraphPad Software Inc., La Jolla, CA, USA) was used to generate graphical representations of flow cytometry data. The gating strategies for flow cytometry analyses are summarised in Supplementary Figure S1.

### 2.7. Whole Blood Immunophenotyping

Whole blood immunophenotyping (WBIP) was carried out on blood samples collected from the macaques enrolled in the ID BCG, aerosol-boosted study, Figure 1c, in order to characterise the γδ T cell profiles in peripheral blood and determine the impact of vaccination. A 50 μL sample of heparinised blood was collected from each animal at each timepoint and incubated for 30 min at room temperature with an antibody cocktail for surface staining, as described above. Erythrocyte contamination was removed using a Cal-lyse reagent kit as per the manufacturer’s instructions (Thermo Fisher Scientific, Swindon, UK). Samples were then fixed in a final concentration of a 4% paraformaldehyde solution (Sigma-Aldrich, Gillingham, UK). Immediately prior to acquisition using an LSRII Fortessa instrument (BD Biosciences, Wokingham, UK), a 50 μL Trucount bead solution (Beckman Coulter, High Wycombe, UK) was added to each sample in order to ascertain the absolute count of each cell population of interest in the blood, as well as their frequencies.

### 2.8. Cytokine and Cytotoxicity Analysis

Intracellular cytokine staining (ICS) assays were utilised to determine the capacity of γδ T cells within the PBMCs or BAL mononuclear cells to produce inflammatory cytokine and degranulation/cytotoxicity markers following BCG vaccination.

Prior to staining, PBMCs or BAL mononuclear cells were incubated overnight with an R10 medium at 37 °C + 5% CO_2_ in the following conditions: without any stimulation; stimulation with BCG at a 1:1 ratio with mononuclear cells (chosen as the result of previous titration); or stimulation with PMA + Ionomycin (positive control). Two hours into the overnight incubation, the protein transport inhibitor Brefeldin-A (Sigma Aldrich, Gillingham, UK) was added at a final concentration of 10 μg/mL, alongside the antibody for degranulation, CD107a (LAMP-1) Pacific Blue (Biolegend, London, UK).

After incubation, cells were washed with the FACS buffer and surface stained following the protocol for surface staining described above, using the antibodies CD3 AF700 (BD Biosciences, Wokingham, UK), TCR Vγ9 FITC, TCR Vδ1 PeCy7 (both Life Technologies, Paisley, UK) and Amine-reactive Live/Dead Fixable Red (Life Technologies, Paisley, UK). Following surface staining, cells were washed and permeabilised by incubating with a cytofix/cytoperm reagent (BD Biosciences, Wokingham, UK) for 15 min at room temperature in the dark. After permeabilization, cells were washed with a Permwash buffer (BD Biosciences, Wokingham, UK) and incubated at room temperature for 30 min with an intracellular staining mix, consisting of the following fluorochrome conjugated antibodies prepared in the Permwash buffer: granzyme B APC, granulysin PE, IFN-γ BV510 (all Biolegend, London, UK); TNF-α (MAb11) BUV395, IL-17 BV711 (both Becton Dickinson UK Limited). Cells were then washed twice with the Permwash buffer, and fixing and flow cytometry was carried out for the surface staining protocol.

### 2.9. Statistics

Raw flow cytometry FCS files were analysed using Flowjo v10.6.2 (BD Biosciences, Wokingham, UK) and data were transferred to Graphpad Prism v9.3.0 (GraphPad Software Inc., La Jolla, CA, USA) for graphical representation and a statistical analysis. Individual values were presented on scatter plots, box plots or histograms, depending on whether the comparison was between groups or timepoints, as well as the number of timepoints shown. Cytotoxicity data are presented using Log2-fold changes. γδ T cell proportion, phenotype and functional responses were compared before and after vaccination using a Wilcoxon-rank test. As one study had an N of 6 (Figure 1a), this test could only determine a maximum strength of association to a *p*-value of 0.031, and therefore the actual *p*-value is expected to be at or lower than 0.031, and therefore designated ‘*p* < 0.040’ throughout. γδ T cell profiles between groups were compared using Mann–Whitney U tests. The main conclusions from the paper are based on findings from pre-planned statistical analyses and therefore *p*-values were not adjusted for multiple comparisons.

To investigate any associations between the γδ T cells and the clinical and health outcomes, Spearman correlation tests were performed using Graphpad Prism v9.3.0 0 (GraphPad Software Inc., La Jolla, CA, USA).

To explore 3-dimentional relationships between phenotypic markers throughout the study, flow cytometry data from six different animals at five different timepoints were concatenated before the t-distributed stochastic neighbour embedding (tSNE) dimensionality reduction was performed using Flowjo v10.6.2 (BD Biosciences, Wokingham, UK). A representative sample of live γδ T cells from each timepoint was taken using the Downsample gate function. tSNE parameters included a perplexity of 30 and 1000 iterations. Cell clusters were manually identified and gated. Heat map overlays represent the level of expression of each marker independently.

## 3. Results

### 3.1. Vδ2 T Cell Populations Expanded in PBMCs after IV BCG Vaccination and Were Associated with a Reduction in TB Pathology

**Figure 2:**
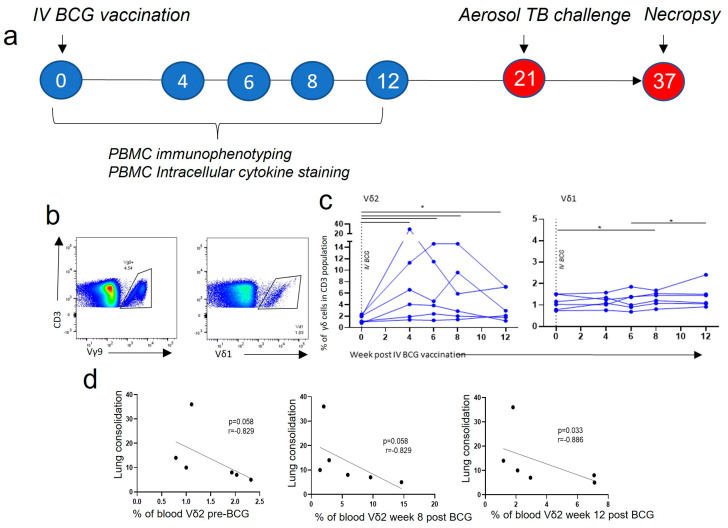
γδ T cells in the circulation after IV BCG vaccination. (**a**) Outline of the experimental study design. (**b**) Gating strategy of Vδ2+ (Vy9+) and Vδ1+ T cells from live CD3+ lymphocytes. (**c**) The proportion of Vδ2 and Vδ1 cells within the CD3+ population in the circulation 4, 6, 8 and 12 weeks after IV BCG vaccination. Data points represent individual animals. Asterisks represent significant differences between indicated timepoints, determined with Wilcoxon tests (*p* < 0.05). Correlations between Vδ2+ (**d**) proportions in the circulation and TB disease and health outcomes were carried out using Spearman correlation tests, with the r and *p*-value shown.

Cryopreserved PBMCs collected from IV BCG-vaccinated animals at 0, 4, 6, 8 and 12 weeks after vaccination were resuscitated. Antibody staining panels (Supplementary Figure S1) were applied and flow cytometry was used to determine how the frequencies of the two main populations of γδ T cells, Vδ2 and Vδ1, changed in the blood after IV BCG vaccination. The proportion of Vδ2 cells within the CD3+ cell population underwent a large and significant expansion 4 weeks after BCG, from a median of 1.5% (range: 0.8–2.3) of the CD3+ population at baseline, to 5.3% (range: 1.4–29.5) of the CD3+ population (*p* < 0.040) (Figure 2c). Furthermore, the Vδ2 cell proportion continued to be of a higher frequency until the last timepoint investigated, 12 weeks post BCG (*p* < 0.040). In contrast, the proportion of Vδ1 cells in the PBMCs showed a small, but significant, increase from baseline at 8 weeks after BCG (*p* < 0.040) (Figure 2c).

Animals from this study received an aerosol *M. tb* challenge 21 weeks after IV BCG vaccination, and clinical outcomes were monitored. TB disease outcomes were measured at necropsy 16 weeks later. Correlation analyses were undertaken to investigate relationships between the immune response post vaccination and clinical measures/disease outcomes. We found that possession of a higher proportion of Vδ2 cells in the PBMCs 12 weeks after IV BCG vaccination was associated with a lower lung consolidation score (Figure 2d) after *M. tb* challenge (*p* = 0.033, r = −0.886). This association was also present at baseline, prior to vaccination, and 8 weeks after vaccination, but did not reach significance (Figure 2d). Unlike Vδ2s, a higher proportion of Vδ1 cells 6 (*p* = 0.044, r = 0.841), 8 (*p* = 0.028, r = 0.899) and 12 (*p* = 0.028, r = 0.899) weeks after IV BCG vaccination was associated with a worse TB pathology score, and a higher Vδ1:Vδ2 ratio was associated with more TB disease (Supplementary Figure S2).

On further examination, responses measured in one animal were markedly different to those made by the rest of the study cohort and it was apparent that the IV BCG did not result in TB protection in this animal. This outlier immune response was characterised by a high proportion of Vδ1 T cells, NK T cells, CD8 T cells and non-classical (CD14+, CD16+) monocytes (Supplementary Figure S2), and may represent interference from common underlying infections, such as cytomegalovirus (CMV) [30].

Overall, the proportion of Vδ2 T cells expanded in the blood after IV BCG vaccination, and a higher proportion of Vδ2 cells was associated with reduced TB disease severity after *M. tb* challenge. We therefore sought to investigate this association in more detail, using phenotypic and functional assays.

### 3.2. Central Memory and Effector Memory Vδ2 Cells Expressing Activation and Homing Markers after IV BCG Vaccination Were Associated with a Reduction in TB Pathology

To investigate potential mechanisms underlying the association between Vδ2 cells and lung pathology after *M. tb* challenge, these cells were examined for phenotypic changes over the time course following IV BCG. Markers of memory, activation, homing and regulation were included in a flow cytometry panel (Supplementary Figure S1).

**Figure 3:**
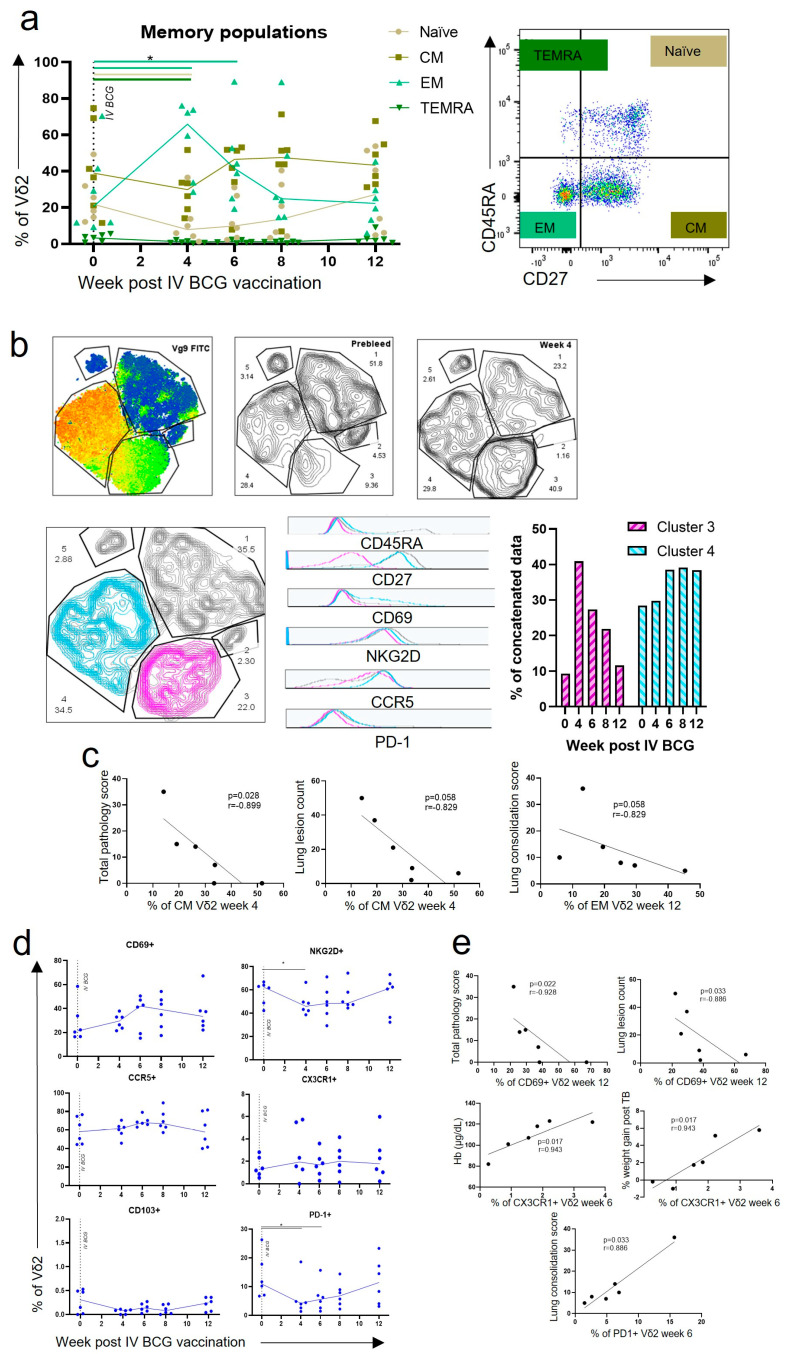
Phenotypic changes of Vδ2 cells after IV BCG vaccination. (**a**) Immunophenotyping flow cytometry assays were utilised to assess changes in memory profiles. Each dot represents one individual. Asterisks represent significant differences between indicated timepoints, determined with Wilcoxon tests (*p* < 0.05). Provided is a representative example of memory profile gating using CD27 and CD45RA expression. (**b**) Phenotypic data from all timepoints were concatenated before dimensionality reduction was performed using tSNE. The heatmap shows the resulting five manually gated clusters. Vδ2 cells are in clusters three and four. The proportion of cells within clusters prior to vaccination and 4 weeks after vaccination is shown. The bar chart shows how the proportion of cells within clusters three and four changes over the course of the vaccination phase. The histograms show the expression of the indicated phenotypic marker in cluster three (pink) and cluster four (blue). (**c**) Spearman correlation tests were used to interrogate associations between memory profiles and TB disease or health measures; *p*-values and r values are shown. (**d**) Immunophenotyping assays were also utilised to assess changes in activation, homing and exhaustion markers. (**e**) Spearman correlations were used to interrogate associations between activation, homing and exhaustion markers and TB disease or health measures. *p*-values and r values are shown.

Changes in the Vδ2 memory phenotype were assessed in PBMCs using CD45RA and CD27 surface markers, as shown in Figure 3a. We found that prior to IV BCG vaccination, the Vδ2 population possessed a high level of central memory (CM) cells expressing CD27+ CD45RA−, a low level of effector memory (EM) cells expressing CD27− CD45RA− and a very low level of terminal effector memory (TEMRA) cells expressing CD45RA+ CD27−. This baseline memory profile is consistent with observations in humans [31,32]. After IV BCG vaccination, the proportion of EM Vδ2 cells underwent a significant expansion, from a median baseline level of 19.3% (range: 11.7–70.4) of Vδ2+ cells, to 65.9% (range: 28.6–76.0) 4 weeks after IV BCG (*p* < 0.040) (Figure 3a). There was also a trend towards an increase in the CM population at weeks six to eight post IV BCG, although this did not reach significance. When the t-distributed stochastic neighbour embedding (tSNE) analysis was performed, γδ T cells separated into five clusters, with Vδ2 cells in cluster three and four (Figure 3b). Cells falling within the phenotypic cluster three at baseline, prior to vaccination, were a small population and increased greatly at week four post IV BCG, whereas phenotypic cluster four was a large population at baseline and increased further between weeks eight and twelve after IV BCG. Examination of phenotypic markers clearly revealed the two clusters to be largely separated using CD27 expression, which relates to their memory profiles. Cluster three cells expressed the profile associated with EM cells, and cluster four expressed the profile associated with CM cells. It was also evident that cluster four, the CM cells, expressed higher levels of CD69 and NKG2D activation markers, as well as more of the regulation marker PD-1. When correlation analyses were performed, possession of a higher proportion of CM Vδ2 cells at week four after IV BCG was associated with a better total pathology score (*p* = 0.028, r = −0.89). There was also a trend for higher EM Vδ2 cells at week 12 after IV BCG, associating with a reduced lung consolidation score, although this did not reach statistical significance (*p* = 0.058, r = −0.829) (Figure 3c).

Examination of Vδ2 phenotypic marker profiles over the 12 weeks after IV BCG vaccination (Figure 3d) revealed non-significant trends towards an increase in CD69 activation marker expression after IV BCG, as well as a trend towards an increase in expression of the homing marker CCR5. The proportion of Vδ2 cells expressing NKG2D and PD-1 significantly decreased at week four post IV BCG (*p* < 0.040 for both), although expression returned to baseline levels by 12 weeks after IV BCG (Figure 3d). Correlation analyses revealed that Vδ2 expression of the CD69 activation marker 12 weeks after IV BCG was associated with a better TB outcome, specifically the total gross pathology score and lung lesion count (*p* = 0.022, r = −0.928, and *p* = 0.033, r = −0.886, respectively). Vδ2 CX3CR1 homing marker expression 6 weeks after IV BCG was also associated with better clinical measures at the end of the study, specifically haemoglobin levels and weight gain following *M. tb* challenge (*p* = 0.017, r = 0.943 for both) (Figure 3e). Conversely, a higher expression of the PD-1 regulatory marker on Vδ2 cells 6 weeks after IV BCG was associated with a higher lung consolidation score (*p* = 0.033, r = 0.886) (Figure 3e).

### 3.3. Vδ2 Cells Produced Inflammatory Cytokines after IV BCG Vaccination, Which Was Associated with A Reduction in TB Pathology

**Figure 4:**
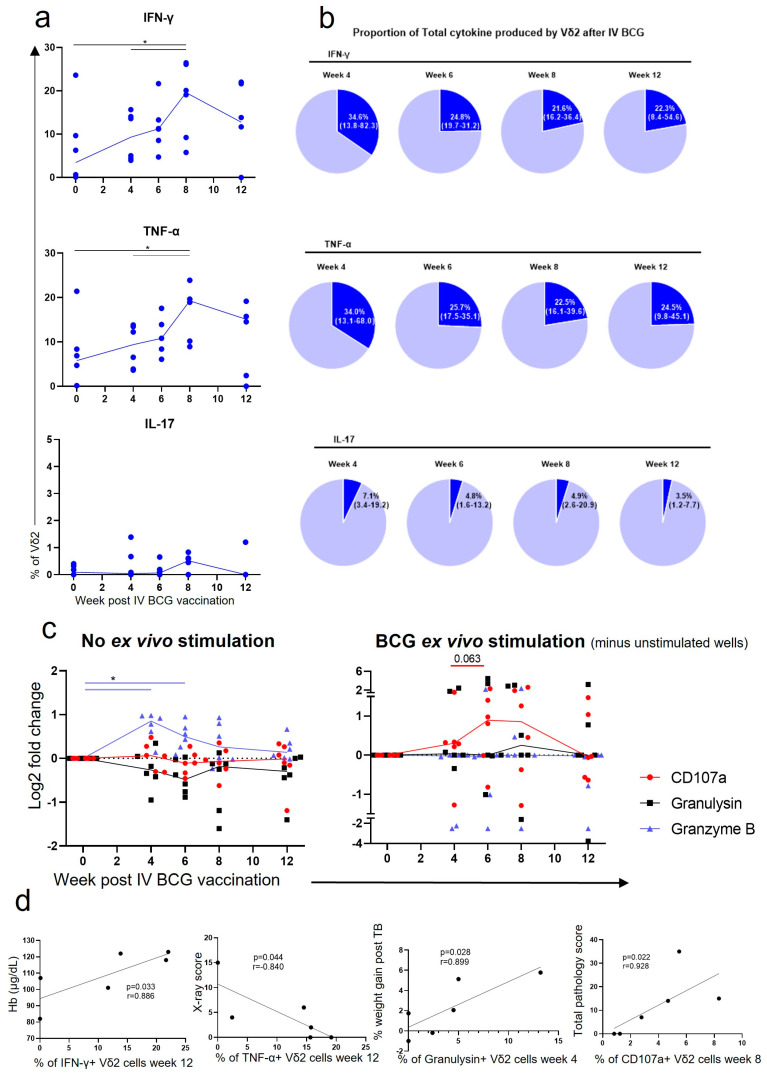
Functional responses of Vδ2 cells after IV BCG vaccination. (**a**) Intracellular cytokine staining (ICS) was utilised to determine the proportion of Vδ2 cells producing IFN-γ, TNF-α or IL-17. Each value represents results from ex vivo BCG-stimulated cells minus results from cells without ex vivo stimulation. (**b**) Vδ2 production of IFN-γ, TNF-α or IL-17 as a proportion of all CD3+ cells producing these cytokines. (**c**) Log2 fold change in the proportion of Vδ2 cells expressing CD107a or producing granulysin or granzyme B, from baseline. Shown are results without any ex vivo stimulation, and with an ex vivo BCG stimulation, minus results from unstimulated cells. Each dot represents results from an individual. Asterisks represent significant differences between indicated timepoints, determined with Wilcoxon tests (*p* < 0.05). (**d**) Spearman correlation tests were carried out to assess whether the proportion of functional Vδ2 cells in the circulation was associated with TB disease or health measures. *p*-values and r values are displayed on the graphs.

Resuscitated PBMCs were stimulated overnight with BCG, intracellular cytokine staining was conducted, and flow cytometry (Supplementary Figure S1) was applied to assess the frequency of BCG-specific cytokine producing cells within each cell population. Prior to vaccination, the proportion of Vδ2 cells capable of producing IFN-γ and TNF-α in response to stimulation with BCG was low, with a median of 3.4% (range: 0.0–23.6) and 5.8% (range: 0.0–21.4), respectively (Figure 4a). The frequency increased rapidly after vaccination and peaked at 8 weeks after IV BCG, with a median of 19.6% (range: 5.8–26.5) and 19.3% (range: 9.0–23.9) Vδ2 cells producing IFN-γ and TNF-α (*p* < 0.040 for both). Figure 4b shows the proportion of Vδ2+ cells within the inflammatory cytokine producing CD3+ population at each timepoint. At 4 weeks after IV BCG, Vδ2 cells account for up to 82.3% (median: 34.6%) of the CD3+ IFN-γ+ cells and up to 68.0% (median: 34.0%) of the CD3+ TNF-α+ cells. This suggests that the Vδ2 cells may be able to produce these cytokines earlier after vaccination than many other CD3+ cells. In contrast, the proportion of Vδ2 cells in the PBMCs that produced IL-17 in response to BCG-specific stimulation did not significantly increase after IV BCG vaccination (Figure 4a), and Vδ2 cells contributed just a small proportion of the CD3+ IL-17+ cells (Figure 4b). When correlation analyses were performed, higher frequencies of BCG antigen-specific Vδ2 cells producing IFN-γ were found to be associated with higher haemoglobin levels measured at necropsy and frequencies of Vδ2 cells producing TNF-α were associated with a lower TB X-ray score (Figure 4d).

Intracellular cytokine staining was used to evaluate the production of cytotoxic molecules within Vδ2 cells. Figure 4 shows the frequency of Vδ2 cells producing cytotoxic molecules in the absence of specific stimulation, together with the frequency of cytotoxic molecule expression following stimulation with BCG (ex vivo) over and above the frequency determined in unstimulated Vδ2 cells. There was a trend towards an increase in the frequency of BCG-specific Vδ2 cells expressing the degranulation marker CD107a after IV BCG vaccination, although this did not reach significance. Similarly, the frequency of Vδ2 cells producing granzyme B independent of BCG stimulation significantly increased at 4 and 6 weeks after IV BCG (*p* < 0.040 for both) relative to those measured before vaccination. Correlation analyses indicated that the possession of more BCG-specific Vδ2 cells producing granulysin 4 weeks after IV BCG was associated with weight gain after *M. tb* challenge, indicating better health. In contrast, more BCG-specific Vδ2 cells expressing CD107a 8 weeks after IV BCG were associated with a higher total pathology score in animals that received aerosol *M. tb* challenge (Figure 4d).

### 3.4. Vδ2 T Cells Did Not Expand in the Blood after ID BCG, but Showed Phenotypic and Functional Changes

**Figure 5:**
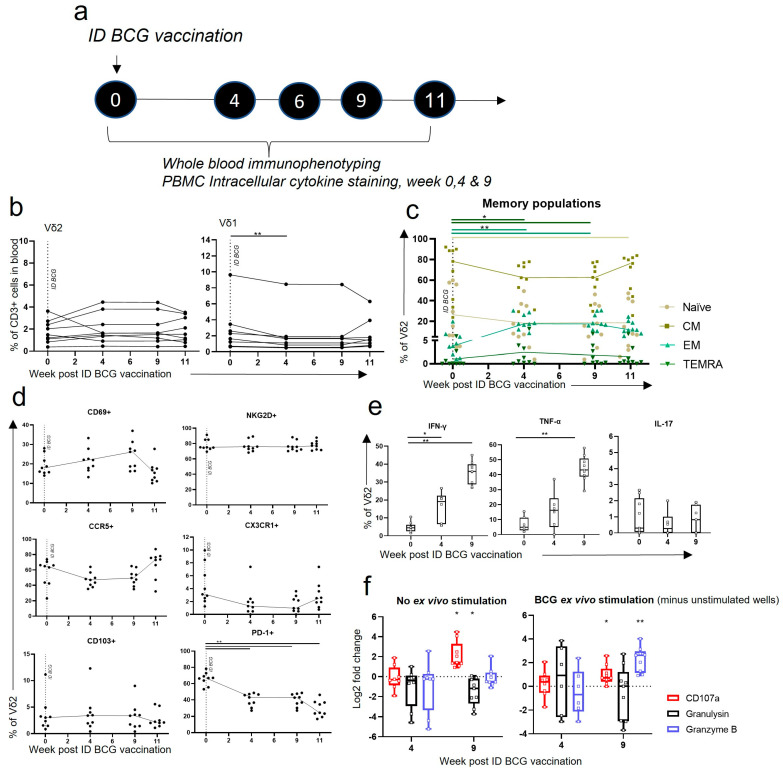
Vδ2 frequency, phenotype and functional profiles after ID BCG vaccination. (**a**) Outline of experimental study design. (**b**–**d**) Whole blood immunophenotyping (WBIP) and (**e**,**f**) intracellular cytokine staining (ICS) conducted on PBMCs prior to and at selected timepoints after an ID BCG vaccination. (**b**) The proportion of Vδ2 and Vδ1 cells in the circulation after ID BCG. (**c**) Memory profiles of Vδ2 cells after ID BCG. (**d**) Expression of activation, homing and exhaustion markers on Vδ2 cells after ID BCG. Each dot represents results from an individual. (**e**) Box plots represent the proportion of Vδ2 cells producing IFN-γ, TNF-α or IL-17 at weeks 0, 4 and 9 after ID BCG. Values represent results from ex vivo BCG-stimulated cells minus results from cells without ex vivo stimulation. (**f**) Log2 fold change in the proportion of Vδ2 cells expressing CD107a or producing granulysin or granzyme B, from baseline. Shown are results without any ex vivo stimulation, and with an ex vivo BCG stimulation, minus results in unstimulated wells. Asterisks represent significant differences between indicated timepoints, or significant change from baseline, determined using Wilcoxon tests, with * = *p* < 0.05 and ** = *p* < 0.01.

To understand whether the changes occurring after IV BCG vaccination in the Vδ2 population were unique to the vaccination route and to compare to standard BCG vaccination, macaques were vaccinated with BCG using intradermal injection and followed for 11 weeks. Cell phenotype and function were explored using whole blood immunophenotyping assays and intracellular cytokine staining (ICS) assays applied to PBMCs at selected timepoints, as described in Figure 5a. Phenotyping and ICS analysis were also undertaken on BAL mononuclear cells at the same timepoints as PBMC ICS (Figure 5a).

Unlike after IV BCG vaccination, we found that the proportion of Vδ2 cells in the CD3+ compartment did not alter significantly after ID BCG vaccination (Figure 5b). There was a small, but significant, decease in Vδ1 cells (*p* = 0.004) 4 weeks post vaccination (Figure 5b). When expression of Vδ2 memory markers, CD27 and CD45RA, was assessed, we found significant changes in the proportions of the memory populations after ID BCG vaccination (Figure 5c). The proportion of EM Vδ2 cells increased relative to pre-vaccination levels at 4 weeks post ID BCG, which was maintained at week 9 (*p* = 0.004 for both), although the extent of the change was markedly less than after IV BCG. Vδ2 TEMRA cells also increased at weeks 4 and 9 (*p* = 0.016 for both) after ID BCG. There was a trend towards a decrease in the Vδ2 CM population after ID BCG, although this did not reach significance (Figure 5c).

The proportion of Vδ2 cells displaying activation markers did not significantly change in the PBMCs after ID BCG vaccination, although, similar to after IV BCG, there was a trend towards an increase in CD69 expression at week nine post ID BCG (Figure 5d). There was a significant decrease in the proportion of Vδ2 cells expressing the PD-1 regulation marker at all timepoints after ID BCG vaccination (*p* < 0.005 for all) relative to pre-vaccination levels. In contrast to IV BCG vaccination, homing marker expression did not significantly change after ID BCG vaccination; however, a trend towards a decrease in CCR5 and CX3CR1 expression was seen (Figure 5).

When ICS assays were undertaken on PBMCs at selected timepoints, we found increased frequencies of BCG-specific IFN-γ-producing Vδ2 cells relative to baseline levels, at week 4 (*p* = 0.032) and 9 (*p* = 0.004) after ID vaccination, as well as TNF-α at week nine (*p* = 0.004) (Figure 5e). The proportion of Vδ2 cells producing these BCG-specific cytokines in the PBMCs was high in comparison to those measured after IV BCG, with a median of 35.9% (range: 26.8–45.1) producing IFN-γ and 43.2% (range: 29.10–57.89) producing TNF-α at week nine post ID BCG. Figure 5f also shows that the frequency of Vδ2 cells expressing the CD107a degranulation marker increased 9 weeks after ID BCG vaccination (*p* = 0.004) relative to baseline levels, and this further increased in response to ex vivo BCG stimulation (*p* = 0.008).

These data indicate that even though Vδ2 cells are not expanded in the circulation after ID BCG, as they are after IV BCG, they respond to vaccination, and show BCG-specific functional responses, specifically the production of IFN-γ and TNF-α and the expression of CD107a. Thus, we hypothesised that investigating the phenotype and function of Vδ2 cells in the lung would provide further insights into the differences caused by the altered vaccination routes, especially given IV BCG protection was demonstrated against aerosol *M. tb* infection.

### 3.5. IV BCG Results in a Higher Proportion of CD69+ CCR5+ Vδ2 Cells in the Lung in Comparison to ID BCG Vaccination

**Figure 6:**
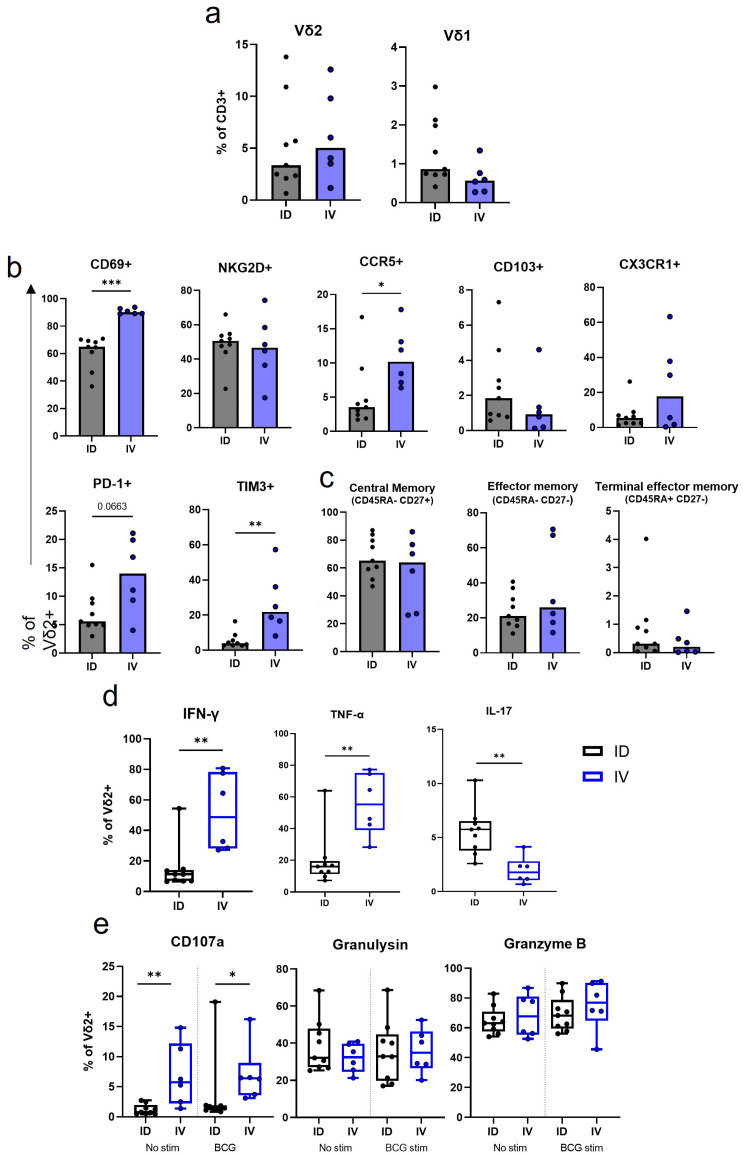
BAL Vδ2 T cell frequency, phenotype and function after ID or IV BCG vaccination. Immunophenotyping and functional flow cytometry panels were applied to BAL mononuclear cells 8 or 9 weeks post IV (blue) or ID (black) BCG vaccination. (**a**) The proportion of BAL Vδ2 cells and Vδ1 cells in the BAL after BCG vaccination. (**b**) The proportion of Vd2 cells expressing activation, homing and exhaustion markers in the BAL. (**c**) The proportion of Vδ2 memory profiles. (**d**) The proportion of BAL Vδ2 cells producing IFN-γ, TNF-α and IL-17 after IV or ID BCG vaccination. Values represent results from ex vivo BCG-stimulated cells minus results from cells without ex vivo stimulation. (**e**) Proportion of Vδ2 cells expressing CD107a, or producing granulysin, or granzyme B. Shown are results without any ex vivo stimulation, and with an ex vivo BCG stimulation, minus results in unstimulated wells. Mann–Whitney U tests were used to test differences between the vaccination routes. A significant *p*-value is denoted by asterisks, with * = *p* < 0.05, ** = *p* < 0.01 and *** = *p* < 0.001.

Bronchoalveolar lavages (BALs) were collected from macaques 8 to 9 weeks after BCG vaccination to coincide with the peak in functional responses identified in the circulation after both IV and ID vaccination strategies. Comparison of Vδ2 cell phenotypes identified in BAL samples collected after IV or ID BCG vaccination revealed that the proportion of Vδ2+ cells did not significantly differ at this timepoint (Figure 6a), whereas differences were observed in the Vδ2 phenotype between the vaccination strategy groups (Figure 6b). A median of 90.1% (range: 89.0–93.9) of the Vδ2 cells recovered from IV BCG-vaccinated macaque BAL samples expressed CD69, significantly more than the BAL Vδ2 cells from ID BCG-vaccinated macaques (*p* = 0.0004). IV BCG BAL Vδ2 cells also showed significantly higher expression of the CCR5 inflammatory homing marker (*p* = 0.026) (Figure 6b). CD69 is an early activation marker thought to prepare cells for tissue residency, and therefore these results may indicate that a high proportion of these BAL Vδ2 cells may traffic to the airways or lung after IV BCG. However, we did not observe a difference in expression of CD103 or CX3CR1 homing/residency markers between the routes at this timepoint. Nor was there any difference in the memory populations represented at this timepoint (Figure 6c).

BAL Vδ2 cells from IV BCG animals showed significantly higher expression of the regulation marker TIM3 (*p* = 0.002), and a trend towards higher expression of PD-1 (*p* = 0.066), than cells from ID BCG animals 8 to 9 weeks after vaccination (Figure 6b). As TB pathology is a result of an unchecked immune system, this negative regulation at sites of infection may be important in limiting pathology.

### 3.6. IV BCG Results in More BCG-Specific Inflammatory Cytokine and Cytotoxic Molecule Producing Vδ2 Cells in the BAL

Cytokine and cytotoxic molecule production by BAL Vδ2 cells was evaluated 8 to 9 weeks after IV or ID BCG vaccination. We found a higher proportion of BCG-specific Vδ2 cells producing IFN-γ and TNF-α in the BAL after IV BCG vaccination than after ID BCG vaccination (*p* = 0.003 for both) (Figure 6d). In comparison to the same timepoint in the blood (Figure 5e), only a small proportion of Vδ2 cells in the BAL produced these cytokines after ID BCG, with a median of 11.3% (range: 6.6–54.4) producing IFN-γ and 16.1% (range: 7.3–64.0) producing TNF-α (Figure 6d). In contrast, after IV BCG vaccination, a higher proportion of BAL Vδ2 cells produced these cytokines than those in the blood, with a median of 48.6% (range: 27.2–80.7) producing IFN-γ and 55.4% (range: 28.4–77.3) producing TNF-α. More IL-17 producing Vδ2 cells were present in the BAL after ID BCG vaccination, than after IV BCG vaccination (*p* = 0.0004) (Figure 6d).

Figure 6e shows the proportion of BAL Vδ2 cells that produced cytotoxic molecules 8 to 9 weeks after IV or ID BCG vaccination. A higher proportion of Vδ2 cells expressing CD107a was found both independent of specific stimulation (background) and in response to stimulation with BCG (beyond background) in the BAL after IV BCG vaccination than after ID BCG vaccination (*p* = 0.005 and *p* = 0.012). This indicates that the BAL Vδ2 cells were degranulating in response to IV BCG vaccination, and this degranulation increased with a further ex vivo BCG exposure. The proportion of Vδ2 cells with intracellular granulysin and granzyme B did not differ between the two vaccination routes.

### 3.7. Aerosol BCG Boosting of ID BCG-Vaccinated Macaques Leads to Changes in BAL Vδ2 Phenotype

Despite the induction of phenotypic and functional changes in the circulation with standard ID BCG vaccination, these did not translate to changes locally in the BAL, which may be important for TB protection. We therefore sought to understand whether an aerosol boost could induce changes in the Vδ2 cells locally in the BAL, as IV BCG vaccination had.

**Figure 7:**
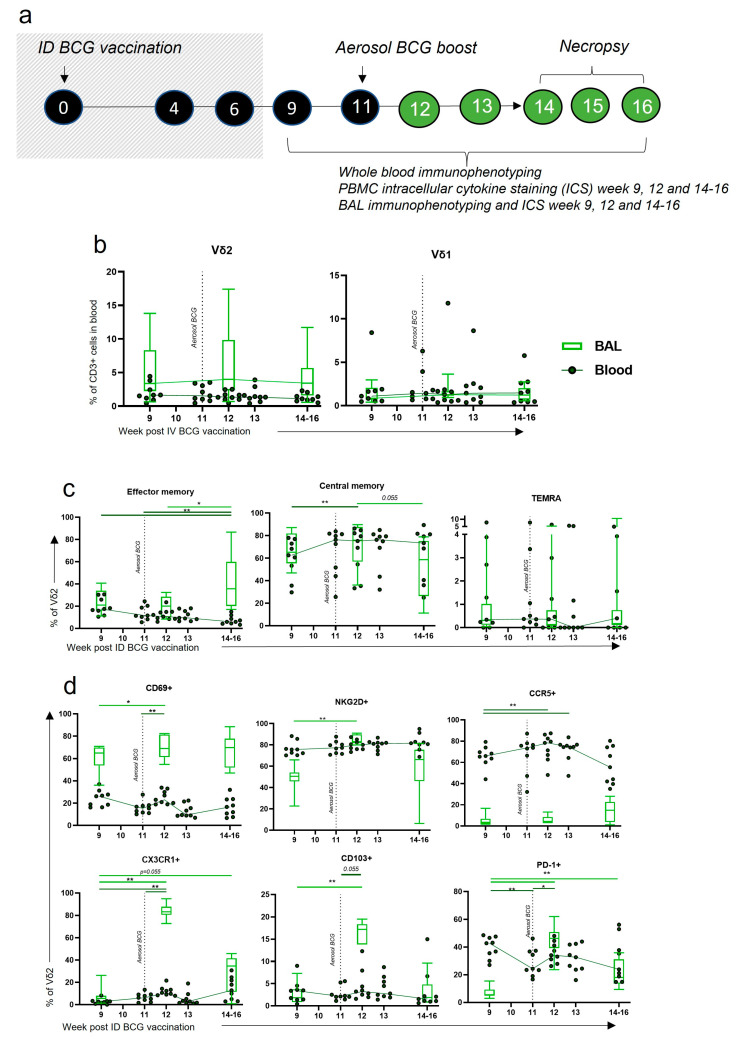
γδ T cell frequency and phenotype after ID BCG + aerosol BCG boost. (**a**) Outline of experimental design, with shaded-out area described previously in Figure 5. Individuals received an aerosol BCG vaccination 11 weeks after initial ID BCG prime and both systemic (dark-green dot plots) and mucosal (light-green box plots). γδ T cell responses were monitored via whole blood immunophenotyping and BAL mononuclear cell immunophenotyping flow cytometry panels, respectively. (**b**) The proportion of Vδ2 and Vδ1 T cells within the live CD3+ population of the blood and BAL prior to and after the aerosol BCG boost. (**c**) Proportion of Vδ2 memory populations. (**d**) The proportion of BAL Vδ2 cells expressing activation, homing and exhaustion markers. Asterisks represent significant differences between indicated timepoints, determined using Wilcoxon tests, with * = *p* < 0.05 and ** = *p* < 0.01.

The cohort of animals used to investigate the Vδ2 cell response to ID BCG vaccination (Figure 5) received an aerosol BCG boost 11 weeks after their initial ID BCG vaccination (Figure 7a) and the phenotype of Vδ2 cells was evaluated (Figure 7b–d) in blood and BAL samples. The proportion of γδ T cells did not significantly change in the blood or BAL in response to the aerosol BCG boost, although there was a trend towards higher proportions of Vδ2 cells in the BAL 1 week post boost (Figure 7b). More Vδ2 cells were identified in the BAL than in the blood. A significant increase in the proportion of Vδ2 cells in the BAL that had an EM profile occurred between 3 and 5 weeks post boost (*p* = 0.027), which corresponded to a decrease in the blood (*p* = 0.004) (Figure 7c). CM Vδ2 in the blood increased 1 week after boost (*p* = 0.012), although levels were increasing prior and may still be a result of the ID BCG prime.

After the aerosol boost vaccination, expression of the CD69 activation marker on Vδ2 cells significantly increased in both the blood and BAL (*p* = 0.004 and *p* = 0.031) (Figure 7d). CD69 expression on Vδ2s circulating in the blood appeared to drop rapidly, but remained high in cells isolated from the BAL. The activation marker NKG2D was highly expressed on Vδ2 cells in the blood throughout the time course but was expressed to a lower degree in the BAL. After the aerosol boost, the proportion of BAL Vδ2 cells expressing NKG2D increased from a median of 50.6% (range: 22.7–66.0) to a median of 83.1% (range: 80.0–91.0) 1 week post boost (*p* = 0.004) (Figure 7d).

Homing marker expression by Vδ2 cells underwent considerable change after the aerosol BCG boost. The proportion of Vδ2 cells expressing CX3CR1 and CD103 in the BAL increased significantly 1 week post boost, from a median of 5.53% (range: 1.3–26.2) to 83.3% (range: 72.8–94.9) (*p* = 0.004), and a median of 1.84% (range: 0.58–7.31) to 17.2% (range: 7.91–19.5) (*p* = 0.004), respectively (Figure 7d). Vδ2 expression of CCR5 in the blood was high and increased further after the aerosol boost (*p* = 0.008). In contrast, CCR5 expression on BAL Vδ2 cells was low, with the suggestion of an upward trend between 3 and 5 weeks post boost. Lastly, 1 week after the aerosol boost, a large increase in the proportion of Vδ2 cells expressing PD-1 in the BAL (*p* = 0.004) was observed together with a smaller increase in the blood (*p* = 0.020).

### 3.8. After Boosting, a Decrease in Vδ2 Functional Responses in the Blood Corresponded to an Increase in Functional Responses in the BAL

**Figure 8:**
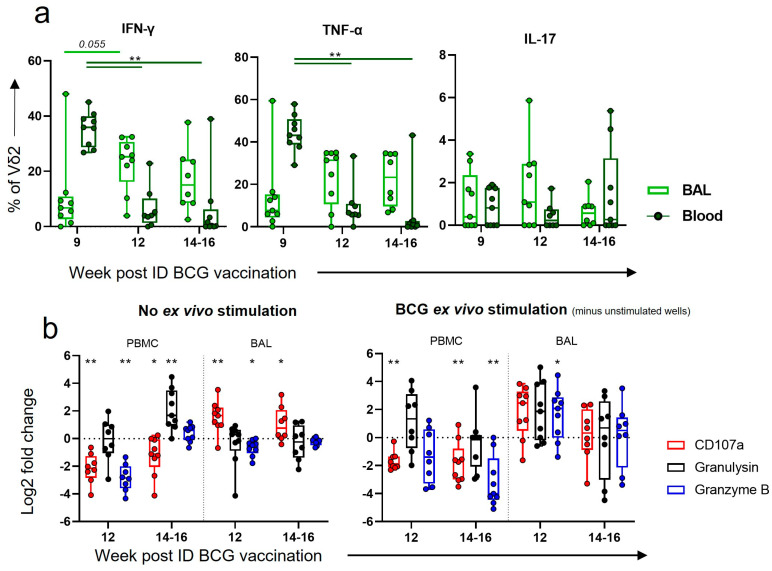
Vδ2 functional responses after ID BCG + aerosol BCG boost. (**a**) Intracellular cytokine staining (ICS) was utilised to determine the proportion of Vδ2 cells producing IFN-γ, TNF-α or IL-17 in the circulation (dark-green box plots) or in the BAL (light-green box plots) prior to and after an aerosol BCG boost. Results are from ex vivo BCG-stimulated wells minus results from wells without ex vivo stimulation. (**b**) Log2 fold changes in the proportion of Vδ2 cells expressing CD107a, or producing granulysin, or granzyme B, from baseline. Shown are results without any ex vivo stimulation, and with an ex vivo BCG stimulation, minus results in unstimulated cells. Asterisks represent significant differences between indicated timepoints, with * = *p* < 0.05 and ** = *p* < 0.01.

As ID BCG induced functional responses in Vδ2 cells in the blood, but did not lead to functional Vδ2 cells in the BAL, we hypothesised that an aerosol BCG boost could direct these functional cells to the airways. We assessed this using intracellular cytokine staining of PBMCs and BAL mononuclear cells prior to the boost (week 9 post ID BCG), 1 week post boost (week 12 post ID BCG) and at necropsy 3 to 5 weeks post boost (14–16 weeks post ID BCG). A substantial decrease in the proportion of IFN-γ- and TNF-α-producing Vδ2 cells in the blood was observed 1 week (*p* = 0.008 for both) and maintained 3 and 5 weeks (*p* = 0.004 and *p* = 0.008) after the boost. In contrast, there appeared to be an increase in the proportion of Vδ2 cells producing these cytokines in the BAL at the same timepoints, although this did not reach statistical significance (Figure 8a).

Examination of cytotoxicity markers after the aerosol BCG boost revealed that the proportion of Vδ2 cells displaying the CD107a degranulation marker significantly decreased in the blood 1 week (*p* = 0.008) and 3 to 5 weeks post boost (*p* = 0.023) (Figure 8b). In contrast, the proportion of Vδ2 cells displaying CD107a in the BAL increased 1 week (*p* = 0.008) after the aerosol boost and there was a trend towards an increase between 3 and 5 weeks post boost (*p* = 0.055). A trend towards a further increase after ex vivo BCG stimulation was also observed.

## 4. Discussion

Utilising the rhesus macaque model of IV BCG, we successfully investigated the role of γδ T cells in the IV BCG vaccine response, and determined that increased Vδ2 responses after vaccination are correlated with a decrease in TB disease severity, as outlined in Figure 2. We were also able to determine key differences in Vδ2 T cell responses between ID and IV vaccination routes by exploring the phenotype and function of these cells in the circulation (Figure 3, Figure 4 and Figure 5) and locally in the BAL (Figure 6). Lastly, we were able to show that an aerosol BCG boost after ID BCG prime can redirect the immune response from the circulation to the BAL (Figure 7 and Figure 8), which may be important for protection from a respiratory *M. tb* infection.

There is a lack of knowledge governing our understanding of what constitutes a protective immune response to *M. tb* infection and TB disease. The paradigm of a largely CD4+ response producing IFN-γ to enhance phagocytic cell activities is an important but limiting model. It is now recognized that there are many other important cell types in the TB response, including γδ T cells, MAIT cells and NK cells, as well as other secreted factors, such as IL-17 and other cytokines, cytotoxic molecules and antibodies, as reviewed by others [23,33,34]. The extent and timing of these responses is also an important determinant of TB outcome, as an extensive cytokine/cytotoxicity response can be protective early in infection, but increase pathology if not controlled [35].

The BCG vaccine given using the standard route of intradermal injection offers limited immunity. However, when given using the IV route in preclinical models, it has been shown to induce far superior protection, even sterilising immunity [11,12]. However, IV BCG delivery may come with safety risks associated with delivering live and replicating pathogenic bacteria to the blood stream, as well as an increase in complications due to the requirements of larger needles [33,36]. Furthermore, there are practicality concerns regarding roll out for human vaccination programs, including the need for more highly trained personnel than ID BCG, as well as the shipment and storage requirements of IV bags [33]. However, there has been a swell of interest in using this route as a means to investigate biomarkers and immune correlates of protection.

### 4.1. Memory Vδ2 T Cells Expressing Activation and Homing Markers Were Associated with Better TB Outcome after IV BCG

We found that Vδ2 T cells expanded in the blood after IV BCG and were associated with less severe TB pathology after *M. tb* challenge (Figure 2). As 4 weeks after IV BCG was our earliest timepoint investigated, it is possible that this expansion occurred even earlier. Previous studies have found an increase in Vδ2 cells in macaque PBMCs between 2 and 5 weeks after IV BCG, with no similar expansion occurring after ID BCG [12,21]. Our study is the first to show that Vδ2 T cells induced after IV BCG have features that correlate with TB outcome.

Vδ2 cells have many properties that may make them an important tool in the arsenal against *M. tb* infection, including rapid non-MHC-dependent activation. The Vδ2 T cell receptor (TCR) recognises small phosphoantigen build-up intracellularly via conformational changes occurring on the Butyrophilin receptors (BTN3A1 intracellularly, then BTN2A1 extracellularly) [37]. This build-up also occurs when there are disruptions to the mevalonate cholesterol synthesis pathway, as in tumour cells and some virally infected cells [37]. Importantly, internalised mycobacteria produce the small phosphoantigen (E)-4-hydroxy-3-methyl-but-2-enyl pyrophosphate (HMBPP) as part of their normal metabolism, and HMBPP has been shown to be a more potent activator of Vδ2 cells (up to 30,000-fold more) than isopentenylpyrophosphate (IPP), which is the equivalent endogenous phosphoantigen [38]. Once activated, Vδ2 cells are potent producers of cytokines and cytotoxicity molecules to directly or indirectly kill cells [39]. Dieli et al. showed that human Vδ2 cells can kill TB-infected macrophages, and this killing was MHC-independent, γδ TCR-dependent and granule-dependent [40]. As well as killing by degranulation, Vδ2 T cells can also induce direct killing through the expression of membrane-bound TNF-family members FasL and TNF-apoptosis-inducing ligand (TRAIL) [37,41], as well as indirectly through the production of inflammatory cytokines and assisting dendritic cell (DC) maturation to stimulate the adaptive immune system [42].

The switch in the Vδ2 memory phenotype towards EM and CM after IV BCG vaccination was associated with a better TB outcome (Figure 3e). There is much evidence for protective roles of EM αβ cells, in both the CD4 and CD8 subsets, in TB disease [22,23,43], yet the evidence for EM γδ T cells is lacking. In humans, EM Vδ2 cells have been shown to be able to produce large amounts of IFN-γ in response to IPP stimulation, but have little proliferation, whereas CM Vδ2 cells have the reverse characteristics [31]. Gioia et al. showed that patients with active TB had considerably fewer EM Vδ2 in circulation, and less IFN-γ-producing cells than healthy controls [44]. In vitro, memory profiles of γδ T cells develop sequentially, from naïve to CM, CM to EM and EM to TEMRA [31]; therefore, high levels of CM Vδ2 cells prior to infection may be important for a rapid Vδ2 EM response. CM Vδ2 cells did not increase in the circulation after ID BCG; instead, an increase in EM and TEMRA profiles was observed. It has long been proposed that the development of long-term central memory CD4 T cells after vaccination is important for TB protection, and that ID BCG largely fails to achieve this, in favour of EM and TEMRA cells [22,45,46,47]. Whether γδ T cells can develop long-term memory is still a matter of debate, yet it has been shown that the memory-like properties of these cells after BCG are sustained for several months [48], and γδ tissue resident populations in other diseases can be long-lasting, as reviewed by Khairallah et al. [49]. It is therefore possible that the development of longer-term γδ CM cells that can rapidly switch to EM is an important difference between IV and ID BCG.

### 4.2. Homing of Functional Cells to Peripheral Sites May Be an Important Part of the Vδ2 IV BCG Response

Our finding of a transient trend towards an increase in CD69 and CCR5 expression by Vδ2 cells in the blood after IV BCG (Figure 3d), followed by the heightened expression of these markers in the BAL compared to after ID BCG (Figure 6b), suggests that circulating Vδ2 cells may travel to the airway. CD69 expression is a marker of early activation, and is also thought to prepare cells for tissue residency, as well as being expressed constitutively on tissue resident T cells in the respiratory tract [50]. CCR5 expression is known to play an important role in targeted migration of immune cells [51], and it has been shown that mice lacking the expression of CCR5 on CD8 cells have a 50% reduction in EM CD8+ T cell migration from pulmonary vascular compartments to interstitial compartments [52].

We also found that IV BCG vaccination led to a high proportion of functional Vδ2 cells in the airways (Figure 6d,e), whereas ID BCG only induced functional cells in the circulation (Figure 5e,f and Figure 6d,e). Induction of local BCG-specific responses may be an important distinguishing factor between these vaccination routes. It has long been established that IFN-γ and TNF-α are involved in the active phase of disease, and, in the absence of true correlates of protection, are often used as a measure of vaccine-induced protection [11,53,54]. Although this is usually thought of in the context of CD4 T cells, it is now known that γδ T cells are also a key producer of inflammatory cytokines after infant and child BCG vaccination [55,56,57,58]. Interestingly, although a lower proportion of Vδ2 cells in the BAL were producing IFN-γ and TNF-α after ID BCG than IV BCG, the opposite was seen for IL-17, suggesting the possibility of distinct pathways being induced between these routes. IL-17 has been shown to be another important inflammatory cytokine in the repertoire against TB, and specifically γδ T cell expression of IL-17 promotes cell recruitment and granuloma formation in the lungs [59,60]. However, it should be noted that excessive IL-17 production may mediate lung immune pathology, as is seen in many autoimmune diseases and tumours [61,62].

The BAL Vδ2 cells were also expressing more CD107a in response to BCG stimulation after IV BCG in comparison to ID BCG. This suggests that these cells are able to degranulate in a memory-like fashion to BCG after IV BCG vaccination, which may be particularly important for the first line of defence against an aerosol *M. tb* infection. Interestingly, expression of this marker in the blood later in the vaccination phase was associated with an increase in pathology (Figure 4d), which may reflect that delayed, systemic cytotoxic responses, as opposed to early, local responses, could be detrimental to TB pathology, as recently reviewed by Chandra et al. [35]. The production of granulysin in response to BCG stimulation early after vaccination was associated with better health after *M. tb* challenge. It has recently been shown that granulysin, in addition to inducing cell death, can contribute to the maturation and migration of DCs, which in turn stimulate the adaptive immune system, including CD4 and CD8 cells [63].

### 4.3. An Aerosol BCG Boost Can Redirect ID BCG Immune Responses to the Airway

We found that boosting with BCG delivered via aerosol route 11 weeks after ID BCG altered the immune response, primarily in the BAL (Figure 7 and Figure 8). This shift was characterised by an increase in Vδ2 EM cells and functional cells identified in the BAL that corresponded to a decrease in the circulation. The Vδ2 cells in the BAL also showed an increase in CD69 and NKG2D expression, as well as a significant, but transient, increase in homing markers (Figure 7d). Altogether, the kinetics of this response indicate that functional cells are moving from the circulation to the airway. It is possible that further transmigration is occurring in the lung compartments between weeks 3 and 5 post boost, given the transient expression of CD103a and CX3CR1 homing markers and the corresponding trend towards a decrease in the proportion of BAL Vδ2 cells as a whole.

Revaccination with BCG in adolescence has been proposed by many as a way to boost the waning BCG immune response. Large Randomised Control Trials (RCTs) have taken place in Brazil, Malawi, Guinea Bissau and South Africa [7,8,9,64], with all failing to find notable differences in the incidence of TB between revaccinated individuals and those without an additional BCG vaccine. In Malawi, revaccinating adolescents and children with ID BCG did not have any effect on TB during 5 to 9 years of follow up, but did provide protection from leprosy [10]. A total of 30 years on from the BCG revaccination in this study, the trial group recently reported no effect on all-cause mortality [64]. In Brazil, a clustered RCT trial took place in two geographically separated cities, and although overall ID BCG revaccination did not impact TB incidence, there was an impact on a subgroup from Salvador [8]. The authors suggested BCG revaccination may have more impact in Salvador than the Manaus because it is further away from the equator and therefore the prevalence of non-tuberculosis mycobacteria is likely to be lower [8]. In the South African study, there was no impact of ID BCG revaccination on tuberculosis incidence, but BCG revaccination reduced the rate of sustained QuantiFERON-TB Gold In-tube assay (QFT) conversion [7], which may reflect a reduction in sustained *M. tb* infection. In all these studies, both the prime and boost were given via the ID route, and it is yet to be determined whether an aerosol BCG boost would improve revaccination efficacy in humans as has been shown in animal studies [11,65].

We and others previously demonstrated that aerosol-delivered BCG is well tolerated in macaques [12,20,66,67]. However, caution is warranted in the delivery of live bacilli to the lung and nasopharyngeal surfaces in humans. Phase I clinical trials centred on this delivery will produce the requisite safety data going forward [68,69]. Furthermore, advances in nebulizer technology, such as the development of portable vibrating mesh nebulisers (VMN) [70], have improved the practicality of a mass aerosol BCG vaccination campaign [20]. We previously showed that vaccinating macaques with ID BCG with an intratracheal mucosal BCG boost 11 weeks later resulted in a reduction in pulmonary disease compared to ID BCG alone [11]. We also previously showed that a single aerosol BCG vaccination resulted in delayed systemic immune responses in comparison to ID BCG [20], highlighting the need for a prime and boost to stimulate both pulmonary and systemic compartments. Darrah et al. did not find any benefit from a simultaneous aerosol and ID BCG vaccination [12], indicating time between the ID prime and aerosol boost is important for the protective kinetics of this response.

A limitation of our study is that our macaques were ID BCG-vaccinated as adults and boosted 11 weeks later. In reality, it may be more feasible for an infant prime and adolescent aerosol boost when protection may be waning. We have now carried out this regime in a small pilot study of macaques, N = 3, and found that, as well as inducing an increase in PPD-specific IFN-γ cells and trained immunity signals, there was also a high proportion of γδ T cells in the BAL [71]. Another limitation is that animals from our ID BCG + aerosol BCG boost study did not go on to receive *M. tb* challenge, and thus the protective efficacy of the changes induced in the BAL cannot be determined. However, as similar changes were occurring to Vδ2 cells in the protective IV BCG response, these results are a promising indicator. Further investigation into this strategy with *M. tb* challenge to determine efficacy is warranted.

Despite protective efficacy, it is unlikely IV BCG will be rolled out in its current form due to safety concerns around the potential consequences of giving a live vaccine intravenously, especially to infants in rural settings. However, application through this route demonstrates the potential that BCG has in eliciting a potent immune response, allowing us to identify new immune correlates of protection and providing us with a benchmark against which we can measure new strategies. Here, we demonstrated the use of both these tools, discovering that Vδ2 T cells are associated with protection in this model, and that ID BCG plus an aerosol BCG boost induces similar changes to these cells in the BAL. These investigations represent pivotal steps in deciphering protective immunological mechanisms against *M. tb,* critical for accelerating the vaccine development pipeline.

## Data Availability

Not applicable.

## Supplementary Materials

The following supporting information can be downloaded at: https://www.mdpi.com/article/10.3390/vaccines11101604/s1, Figure S1: Flow cytometry gating strategies. (a) Gating strategy for Vδ1 and Vδ2 T cells. Lymphocytes were selected from an FSC area vs. SSC area dot plot, and doublets were removed with an FSS area vs. FSC height dot plot. T cells were gated on using their expression of CD3 and dead cells were removed with Amine-reactive Live/Dead Fixable Red viability cell stain (Life Technologies, Paisley, UK). γδ T cells could then be selected from the CD3+ population using antibodies against the Vγ9 portion of the TCR for Vδ2 cells and the Vδ1 portion of the TCR for Vδ1 cells. (b) Gating strategy for phenotyping of Vδ2 T cells. The Vδ2 T cells were selected with the Vγ9 antibody and could be separated out into memory populations using antibodies against CD27 and CD45RA. From the Vδ2 + population, activation, homing and exhaustion marker expression could also be examined using antibodies against CD69, NKG2D, CD103, CX3CR1, CCR5, PD-1 and TIM3. Fluorescence minus one (FMO) controls were utilised to select the appropriate gate. (c) Gating strategy for Vd2 functional markers. Live CD3+ Vd2+ cells were selected and antibodies against the functional markers IFN-γ, TNF-α, IL-17, CD107a, granzyme B and granulysin were utilised to select for cells producing/expressing these markers. FMO controls were utilised to select the appropriate gate; Figure S2: The association between Vδ1 cells and greater TB disease may be due to an outlier. (a) Associations between the proportion of Vδ1 cells within the CD3+ compartment in the circulation after IV BCG vaccination, and TB disease outcomes. (b) Associations between the Vδ1:Vδ2 ratio in the circulation and TB disease outcomes. Associations were measured using Spearman correlation tests, and the r and *p*-value is shown on the graphs. (c–e) Other immune cell populations after IV BCG vaccination were investigated using flow cytometry, including CD4+, CD8+ and NK T cells from the CD3+ population (c); NK cells from the CD3− population (d); and CD14+ CD16+ (non-classical) and CD14+ CD16− (classical) monocytes from live cells (e). (f) Vδ1 and Vδ2 proportions, with the outlier individual noted in red. Asterisk indicates a statistically significant difference between the timepoints indicated (*p* < 0.05). Individual dots throughout represent individuals, and the red dot is the same outlier individual, S35.
