# Supplementary figures and images for: IV BCG Vaccination and Aerosol BCG Revaccination Induce Mycobacteria-Responsive γδ T Cells Associated with Protective Efficacy against M. tb Challenge
Source: Vaccines (Basel). 2023 Oct 17;11(10):1604. doi: 10.3390/vaccines11101604 (PMC10611416; doi:10.3390/vaccines11101604)

Fig S1

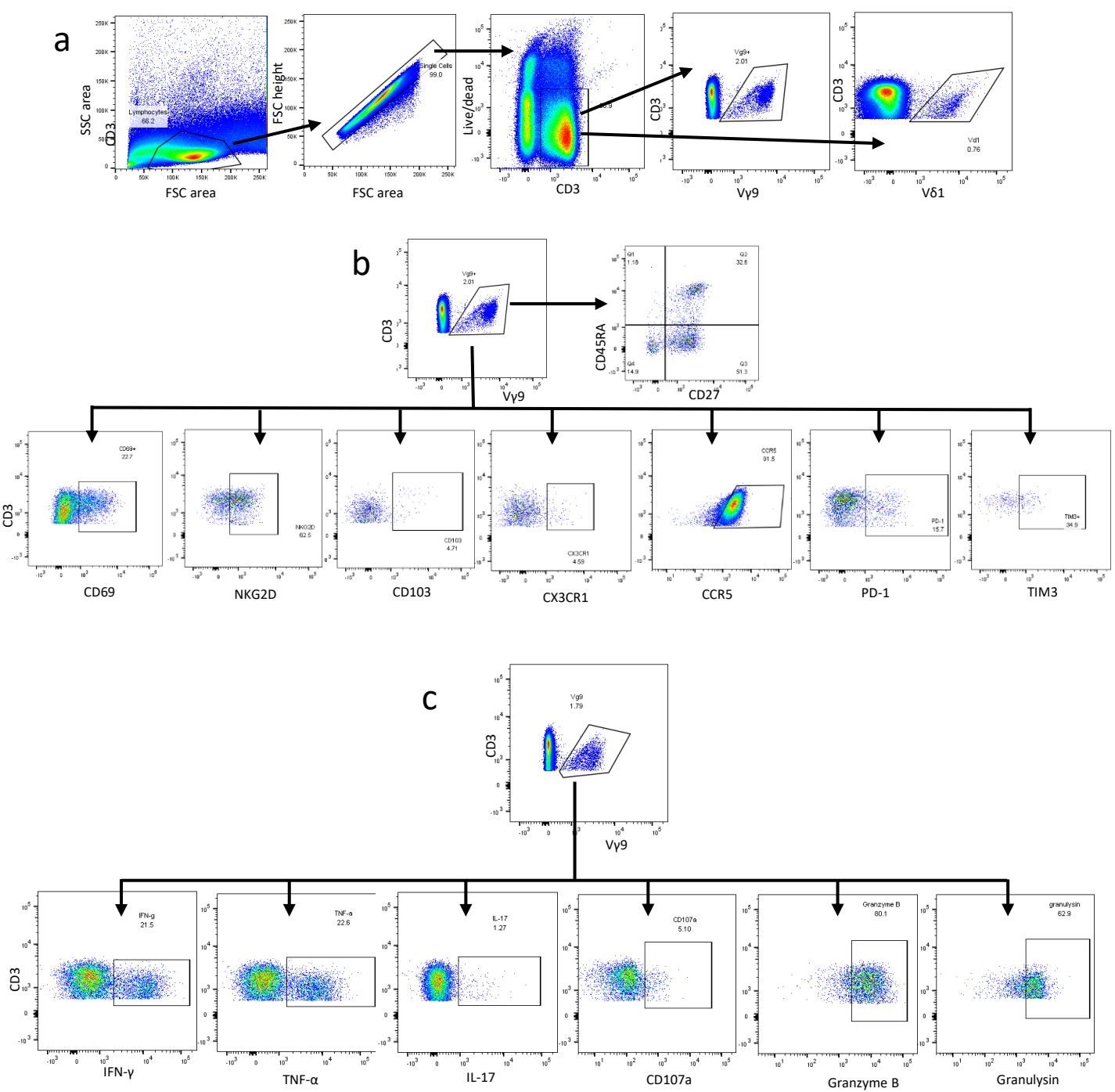

Fig S2

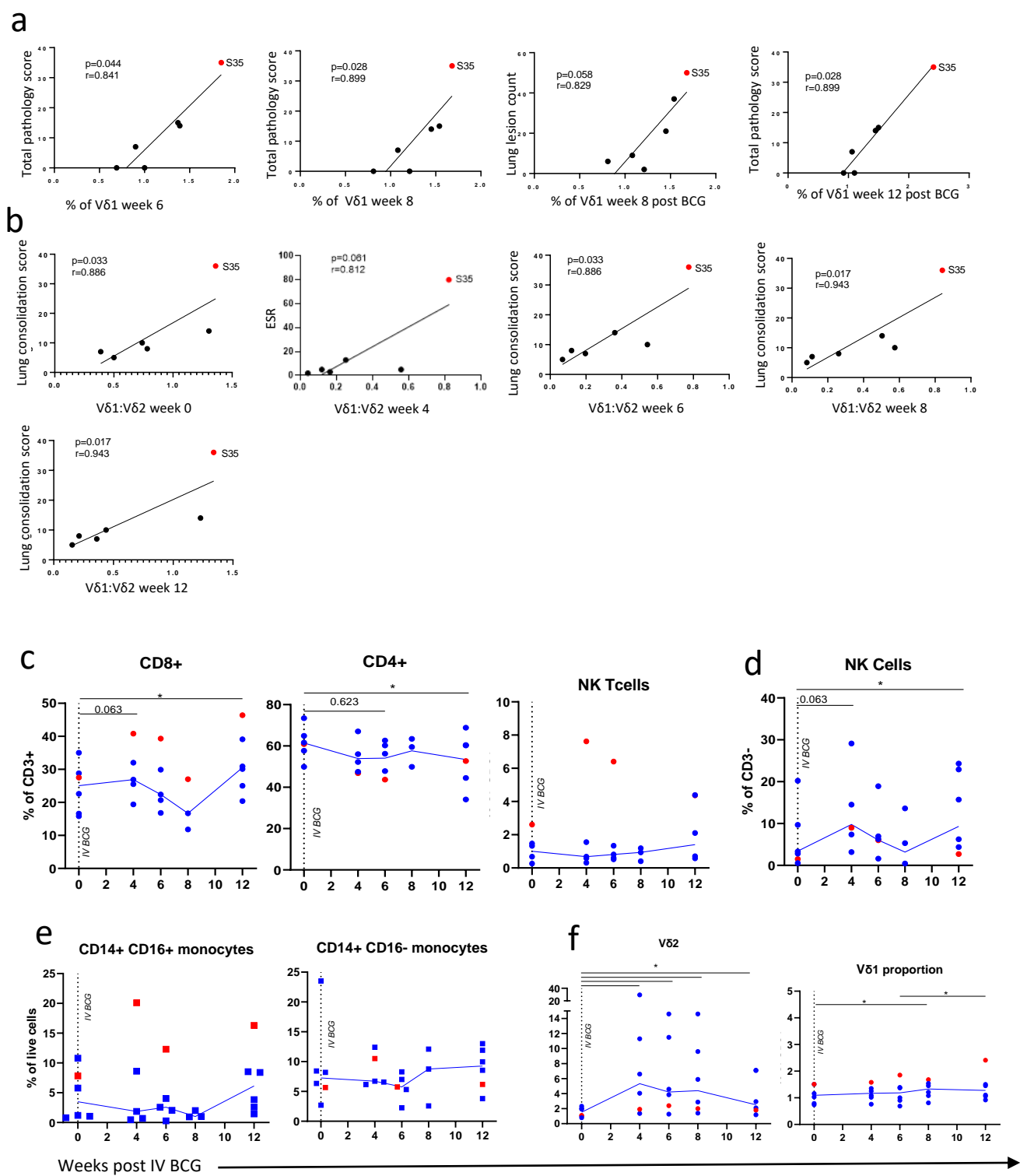

Supplement: Supplementary file 1 [file vaccines-11-01604-s001.zip › vaccines-2627188-supplementary.pdf]
